# A Ten-Year Trend of Antimicrobial Resistance in *Pseudomonas aeruginosa* From United States Medical Centers: Results From the INFORM Surveillance Program (2015–2024)

**DOI:** 10.1093/ofid/ofag504

**Published:** 2026-08-14

**Authors:** Helio S Sader, Rodrigo E Mendes, John Lock, Krisztina M Papp-Wallace, Mariana Castanheira

**Affiliations:** Element Iowa City (JMI Laboratories), North Liberty, Iowa, USA; Element Iowa City (JMI Laboratories), North Liberty, Iowa, USA; AbbVie, Chicago, Illinois, USA; Element Iowa City (JMI Laboratories), North Liberty, Iowa, USA; Element Iowa City (JMI Laboratories), North Liberty, Iowa, USA

**Keywords:** ceftazidime-avibactam, ceftolozane-tazobactam, multidrug-resistant, pneumonia, pseudomonas aeruginosa

## Abstract

We evaluated 14 304 *Pseudomonas aeruginosa* isolates from 56 US medical centers (2015**–**2024). Ceftazidime-avibactam, ceftolozane-tazobactam, and imipenem-relebactam were active against 97.0%**–**97.9% of isolates overall. Piperacillin-tazobactam and ceftazidime activities decreased slightly during the study period, whereas the activities of meropenem, levofloxacin, and tobramycin improved since 2017.

## Teaser

Susceptibility of P. aeruginosa to ceftazidime, meropenem, and piperacillin-tazobactam has oscillated between 78.0% and 83.0% in the last 30 years with no clear tendencies toward increasing or decreasing. Ceftazidime-avibactam, ceftolozane-tazobactam, and imipenem-relebactam remain very active against this organism (97.0-97.9% susceptible).

Antimicrobial treatment of *Pseudomonas aeruginosa* infections represents an important challenge for clinicians. Immediate initiation of appropriate therapy is critical to improve clinical outcomes and reduce morbidity and mortality [1]. However, selection of empiric therapy is difficult as this organism is usually resistant to many antibiotics commonly used to treat gram-negative infections and has the ability acquire a broad array of resistance mechanisms [2].

Resistance to first-line agents varies widely by geographic region and among medical centers within a region. Thus, local antibiograms and data from regional surveillance programs are extremely valuable to guide empiric therapy [2]. The Infectious Diseases Society of America (IDSA) guidance currently recommends the use of newer β-lactam/β-lactamase inhibitor combinations (BLICs), such as ceftazidime-avibactam, ceftolozane-tazobactam, and imipenem-relebactam, for treatment of infections due to difficult-to-treat (DTR) *P. aeruginosa*, defined as an isolate resistant to all first-line agents. These 3 BLICs have shown to be highly active against *P. aeruginosa*, but data from surveillance programs on the in vitro activity and spectrum of these compounds against multidrug-resistant (MDR) and DTR *P. aeruginosa* are limited [3–5]. In this investigation, we evaluated the antimicrobial susceptibility of *P. aeruginosa* isolates collected from US medical centers in 2015**–**2024.

## METHODS

A total of 14 304 *P. aeruginosa* isolates (1/patient) were consecutively collected from 56 US medical centers in 2015**–**2024 as part of the International Network For Optimal Resistance Monitoring (INFORM) program [6]. The participant centers were mostly tertiary hospitals and were in 33 states from all 9 US Census Divisions, and the number of centers in each Census Division ranged from 4 in the Mountain Division to 10 in the East North Central Division. The participating medical centers followed a common protocol that required the gathering of a certain number of consecutive patient unique bacterial isolates (any species) from designated infection types during a certain period of the year. Only bacterial isolates determined to be significant by local criteria as the reported probable cause of infection were included in the study, and only medical centers that contributed to isolates for ≥8 years were included in this investigation.

Isolates were susceptibility tested by Clinical and Laboratory Standards Institute (CLSI) M07 [7] broth microdilution methods at a monitoring laboratory (Element Iowa City [JMI Laboratories], North Liberty, IA, USA), and susceptibility results were stratified by year and infection type. Imipenem-relebactam was included in the program in 2020. All other antimicrobials were tested during the entire period of the investigation (2015**–**2024). Minimum inhibitory concentration (MIC) values were interpreted according to CLSI and/or US Food and Drug Administration (FDA) breakpoints, when available [8, 9]. European Committee on Antimicrobial Susceptibility Testing (EUCAST) breakpoints were applied for meropenem-vaborbactam for comparison purposes as this agent is not approved to treat *P. aeruginosa* infections in the United States and breakpoints have not been established by the FDA or CLSI for this drug–organism combination [10].

MDR was defined as nonsusceptibility (intermediate susceptibility or resistance) to ≥3 antimicrobial classes [11, 12]. The antimicrobial classes, representative agents, and corresponding interpretive criteria for nonsusceptibility were (1) anti-*Pseudomonas* cephalosporins and monobactams (ceftazidime [≥16 mg/L], cefepime [≥16 mg/L], and aztreonam [≥16 mg/L]); (2) carbapenems (meropenem [≥4 mg/L] and imipenem [≥4 mg/L]); (3) old BLICs (piperacillin/tazobactam [≥32/4 mg/L]); (4) new BLICs (ceftazidime-avibactam [≥16/4 mg/L], ceftolozane-tazobactam [≥8/4 mg/L], and imipenem-relebactam [≥4/4 mg/L]); (5) aminoglycosides (tobramycin [≥2 mg/L]); (6) fluoroquinolones (levofloxacin [≥2 mg/L] and ciprofloxacin [≥1 mg/L]); and (7) lipopeptides (colistin [≥4 mg/L]). DTR resistance was defined as nonsusceptibility to ceftazidime, cefepime, piperacillin-tazobactam, imipenem, meropenem, levofloxacin, and ciprofloxacin [3].

## RESULTS

**Figure 1.**
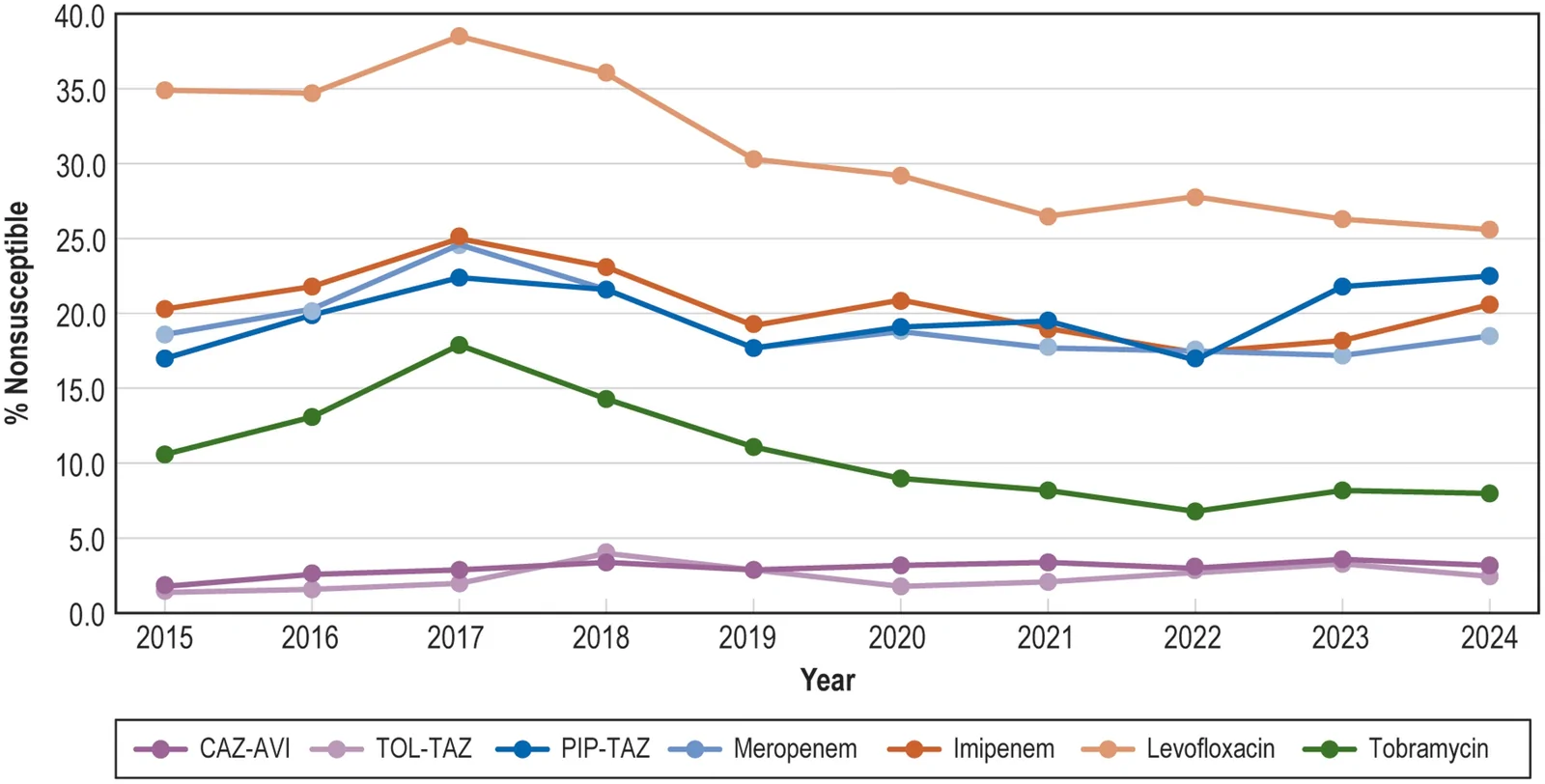
Frequency of *P. aeruginosa* nonsusceptibility to selected agents. Abbreviations: CAZ-AVI, ceftazidime-avibactam; PIP-TAZ, piperacillin-tazobactam; TOL-TAZ, ceftolozane-tazobactam.

The most active compounds overall were ceftazidime-avibactam (MIC_50/90_, 2/4 mg/L; 97.0% susceptible), ceftolozane-tazobactam (MIC_50/90_, 0.5/2 mg/L; 97.6% susceptible), and imipenem-relebactam (MIC_50/90_, 0.25/1 mg/L; 97.9% susceptible) (Table 1). The frequencies of isolates not susceptible to ceftazidime-avibactam and ceftolozane-tazobactam increased slightly from 1.8% and 1.4% in 2015 to 3.2% and 2.5% in 2024, respectively. Nonsusceptibility to imipenem-relebactam decreased slightly from 2.8% in 2020 to 1.3% in 2024 (Table 1, Figure 1).

**Table 1.** Overall Antimicrobial Susceptibility of *P. aeruginosa* (2015–2024)

Antimicrobial Agent	MIC, mg/L		% Susceptible (No. of Isolates)				
	MIC_50_	MIC_90_	All Isolates	2015	2024	MDR	DTR
			(14 304)	(1354)	(1121)	(2459)	(568)
Ceftazidime-avibactam	2	4	97.0	98.2	96.8	83.0	65.3
Ceftolozane-tazobactam	0.5	2	97.6	98.6	97.5	85.9	71.9
Imipenem-relebactam^a^	0.25	1	97.9	^a^	98.7	86.9	64.8
Meropenem-vaborbactam^b^	0.5	8	91.6^b^	92.7^b^	92.6^b^	58.2	0.0
Piperacillin-tazobactam	4	>64	80.2	83.0	77.4	18.5	0.0
Meropenem	0.5	8	80.6	81.4	81.4	22.5	0.0
Imipenem	1	8	79.3	79.7	79.4	26.4	0.0
Ceftazidime	2	32	84.2	86.6	82.2	33.3	0.0
Cefepime	2	16	85.3	88.3	84.2	30.5	0.0
Levofloxacin	0.5	>4	68.7	65.1	74.6	15.6	0.0
Tobramycin	0.5	2	89.0	89.4	92.0	56.5	46.5

Abbreviations: CLSI, Clinical and Laboratory Standard Institute; DTR, difficult to treat; EUCAST, European Committee on Antimicrobial Susceptibility Testing; FDA, Food and Drug Administration; MDR, multidrug resistant; MIC, minimum inhibitory concentration.

^a^Imipenem-relebactam tested only from 2020 to 2024, and susceptibility in 2020 was 97.2%.

^b^Meropenem-vaborbactam is not approved by the US FDA for treatment of *P. aeruginosa* infections; susceptibility is based on EUCAST criteria (susceptible at ≤8 mg/L) [11].

Piperacillin-tazobactam was active against 80.2% of isolates overall (MIC_50/90_, 4/>64 mg/L), and the percentage of isolates not susceptible to this antimicrobial increased from 17.0% in 2015 to 22.6% in 2024. Similarly, nonsusceptibility to ceftazidime (MIC_50/90_, 2/32 mg/L; 84.2% susceptible) increased from 13.4% in 2015 to 17.8% in 2024 (Table 1, Figure 1). Nonsusceptibility to meropenem, imipenem, levofloxacin, and tobramycin increased from 2015 to 2017 and then decreased progressively until 2023 or 2024 (Figure 1). Notably, nonsusceptibility to levofloxacin decreased from 34.9% in 2015 to 25.4% in 2024 (Figure 1).

Overall, 17.2% of isolates were categorized as MDR and 4.0% as DTR. The frequency of MDR and DTR isolates increased until 2017, decreased from 2017 to 2022 or 2023, and then increased again in 2024 (Supplementary Figure 1). The percentage of isolates with an MDR phenotype increased from 15.8% in 2015 to 22.8% in 2017 and then decreased to 16.0% in 2024, with the lowest value in 2022 (12.9%). Ceftazidime-avibactam was active against 83.0% of MDR (MIC_50/90_, 4/16 mg/L) and 65.3% of DTR isolates (MIC_50/90_, 8/32 mg/L). Ceftolozane-tazobactam was active against 85.9% of MDR (MIC_50/90_, 2/8 mg/L) and 71.9% of DTR isolates (MIC_50/90_, 4/>16 mg/L). Imipenem-relebactam was active against 86.9% of MDR (MIC_50/90_, 1/4 mg/L) and 64.8% of DTR isolates (MIC_50/90_, 2/8 mg/L) (Table 1).

The antimicrobial susceptibility of isolates stratified by US Census Divisions is displayed in Supplementary Table 1. Susceptibility rates were lowest in the Middle Atlantic Division, with participant centers in New Jersey, New York, and Pennsylvania. Susceptibility rates were also generally lower in the East South Central (Alabama, Mississippi, and Tennessee), West South Central (Arkansas, Louisiana, Oklahoma, and Texas), and Mountain (California, Oregon, and Washington state) Divisions compared with other divisions. Moreover, the highest susceptibility rates were generally observed in the West North Central Division (Iowa, Kansas, Minnesota, Missouri, Nebraska, North Dakota, and South Dakota) (Supplementary Table 1).

Cross-resistance between new BLICs when testing the entire collection of *P. aeruginosa* as well as the DTR subset is shown in Supplementary Table 2. Ceftazidime-avibactam retained in vitro activity against 37.1% and 53.1% of isolates not susceptible to ceftolozane-tazobactam and imipenem-relebactam, respectively, and 29.8% and 36.6% of DTR isolates not susceptible to ceftolozane-tazobactam and imipenem-relebactam, respectively. Ceftolozane-tazobactam was active against 41.0% and 43.9% of DTR isolates not susceptible to ceftazidime-avibactam and imipenem-relebactam, respectively. Likewise, imipenem-relebactam remained active against 48.0% and 36.1% of DTR isolates not susceptible to ceftazidime-avibactam and ceftolozane-tazobactam, respectively (Supplementary Table 2).

Antimicrobial susceptibility of isolates stratified by infection type is shown in Supplementary Table 3. In general, susceptibility rates were lower among isolates from pneumonia compared with other infections. Susceptibility to ceftazidime-avibactam was 96.4% against isolates from patients with pneumonia and 97.4% to 98.2% against isolates from patients with other infections. Susceptibility to piperacillin-tazobactam was 76.7% against isolates from patients with pneumonia and 83.8% to 85.8% against isolates from patients with other infection types (Supplementary Table 3).

## DISCUSSION

The SENTRY Program has been monitoring the antimicrobial susceptibility of *P. aeruginosa* worldwide since 1997. The geographic and temporal trends in resistant phenotypes of *P. aeruginosa* during the initial 20 years of the SENTRY Program (1997**–**2016) showed that the susceptibility of *P. aeruginosa* to piperacillin-tazobactam and ceftazidime increased slightly in the period of 1997**–**2016 in North America, whereas susceptibility to meropenem showed a minor decrease in the same time frame [13]. When comparing the results of this investigation with those reported by Shortridge et al. for North America, we observe that susceptibility to piperacillin-tazobactam and ceftazidime increased between 1997 and 2016 [13] and then decreased during this investigation (between 2015 and 2024), whereas susceptibility to meropenem oscillated markedly from a high of 85.2% in 1997–2000 to a low of 75.4% in 2017, but remained stable around 82.0% (81.4%**–**82.5%) during the last 6 years (2019**–**2024) of this investigation. Moreover, the frequency of MDR organisms also remained stable in the 2019**–**2024 period (Supplementary Figure 1).

Additionally, our results showed that the BLICs ceftazidime-avibactam and ceftolozane-tazobactam remain very active against *P. aeruginosa* in US medical centers even after >10 years of clinical use and show minimal decrease in activity during the 10-year period of the investigation [14]. Moreover, imipenem-relebactam showed antipseudomonal activity similar to ceftazidime-avibactam and ceftolozane-tazobactam and represents a valuable addition to the arsenal of drugs to treat MDR *P. aeruginosa.* Cefiderocol has also shown good activity and broad spectrum against *P. aeruginosa,* and the fact that it was not tested represents an important limitation of this investigation.

In summary, the results of this investigation coupled with previous publications from our group [5, 13, 14] indicate that the susceptibility of *P. aeruginosa* to ceftazidime, meropenem, and piperacillin-tazobactam in US medical centers has oscillated. Susceptibility rates for piperacillin-tazobactam have been between 77.0% and 83.0% in the last 30 years (since 1997) with no clear tendencies toward increasing or decreasing. The BLICs ceftazidime-avibactam, ceftolozane-tazobactam, and imipenem-relebactam remain very active against this organism.

## Supplementary Material

ofag504_Supplementary_Data

## Supplementary Data

Supplementary materials are available at *Open Forum Infectious Diseases* online. Consisting of data provided by the authors to benefit the reader, the posted materials are not copyedited and are the sole responsibility of the authors, so questions or comments should be addressed to the corresponding author.
