# Supplementary material for: A Ten-Year Trend of Antimicrobial Resistance in Pseudomonas aeruginosa From United States Medical Centers: Results From the INFORM Surveillance Program (2015–2024)
Source: Open Forum Infect Dis. 2026 Aug 14;13(8):ofag504. doi: 10.1093/ofid/ofag504 (PMC13507311; doi:10.1093/ofid/ofag504)
Supplement: Supplementary file 1 [file ofag504_supplementary_data.zip › OFID-D-26-00418R1 Supplemental Material.docx]

**Table S1**. Antimicrobial susceptibility of *P. aeruginosa* stratified by US Census Division (2015-2024)

|  | **% Susceptible (no. tested) by US Census Division** | | | | | | | | |
| --- | --- | --- | --- | --- | --- | --- | --- | --- | --- |
|  | **New England** | **Middle Atlantic** | **East North Central** | **West North Central** | **South Atlantic** | **East South Central** | **West South Central** | **Mountain** | **Pacific** |
| **Antimicrobial agent** | **(1,063)** | **(2,544)** | **(2,048)** | **(1,360)** | **(2,169)** | **(1,130)** | **(1,691)** | **(1,045)** | **(1,254)** |
| Ceftazidime-avibactam | 97.4 | 95.2 | 98.2 | 98.6 | 98.0 | 97.6 | 96.1 | 96.0 | 96.5 |
| Ceftolozane-tazobactam | 97.1 | 95.6 | 98.1 | 99.3 | 98.2 | 97.2 | 97.6 | 96.6 | 95.9 |
| Imipenem-relebactam^a^ | 98.1 | 96.4 | 98.7 | 99.8 | 99.0 | 99.7 | 97.1 | 97.2 | 97.2 |
| Meropenem-vaborbactam^b^ | 94.5 | 87.4 | 94.7 | 97.9 | 95.2 | 94.0 | 88.5 | 92.3 | 94.0 |
| Piperacillin-tazobactam | 81.2 | 72.5 | 84.4 | 87.3 | 83.0 | 78.8 | 78.5 | 78.5 | 80.1 |
| Meropenem | 84.9 | 71.4 | 84.3 | 87.4 | 86.4 | 78.7 | 75.7 | 77.5 | 83.2 |
| Imipenem | 83.2 | 69.4 | 82.9 | 86.1 | 85.0 | 78.7 | 75.6 | 74.5 | 82.4 |
| Ceftazidime | 84.6 | 77.3 | 88.3 | 90.2 | 87.0 | 82.7 | 82.7 | 82.3 | 84.3 |
| Cefepime | 84.9 | 79.8 | 88.8 | 89.4 | 88.3 | 83.6 | 83.3 | 84.5 | 86.3 |
| Levofloxacin | 69.1 | 65.0 | 73.4 | 72.1 | 72.0 | 64.2 | 66.6 | 67.0 | 66.7 |
| Tobramycin | 87.9 | 88.0 | 89.5 | 88.5 | 90.3 | 86.6 | 89.7 | 87.9 | 91.0 |

^a^ Imipenem-relebactam tested only from 2020 to 2024.
^b^ Meropenem-vaborbactam is not approved by the US FDA for treatment of *P. aeruginosa* infections; susceptibility based on EUCAST criteria (susceptible at ≤8 mg/L) [11].

**Table S2**. Cross resistance rates among β-lactamases inhibitor combinations

|  | **% Susceptible by resistance phenotype (no. of isolates)** | | |
| --- | --- | --- | --- |
| **Antimicrobial agent / organism group** | **Ceftazidime-avibactam nonsusceptible** | **Ceftolozane-tazobactam nonsusceptible** | **Imipenem-relebactam nonsusceptible** |
| All isolates | (430) | (307) | (96) |
| Ceftazidime-avibactam | 0.0 | 37.1 | 53.1 |
| Ceftolozane-tazobactam | 46.2 | 0.0 | 55.8 |
| Imipenem-relebactam | 73.1 | 68.9 | 0.0 |
| DTR | (197) | (131) | (41) |
| Ceftazidime-avibactam | 0.0 | 29.8 | 36.6 |
| Ceftolozane-tazobactam | 41.0 | 0.0 | 43.9 |
| Imipenem-relebactam | 48.0 | 36.1 | 0.0 |

Abbreviations: DTR, difficult-to-treat resistant.

**Table S3.** Antimicrobial susceptibility of *P. aeruginosa* isolates stratified by infection type

|  | **% Susceptible (no. of isolates)** | | | | | |
| --- | --- | --- | --- | --- | --- | --- |
| **Antimicrobial agent** | **Pneumonia  (n=7,985)** | **BSI  (n=1,538)** | **SSSI  (n=2,458)** | **UTI  (n=1,571)** | **Others  (n=752)** | **All  (n=14,302)** |
| Ceftazidime-avibactam | 96.4 | 97.7 | 97.4 | 98.2 | 98.0 | 97.0 |
| Ceftolozane-tazobactam | 96.4 | 97.7 | 98.1 | 99.0 | 98.4 | 97.6 |
| Imipenem-relebactam^a^ | 97.7 | 98.5 | 98.2 | 98.0 | 99.6 | 97.9 |
| Meropenem-vaborbactam^b^ | 91.3 | 95.6 | 92.3 | 94.5 | 95.7 | 91.6^b^ |
| Piperacillin-tazobactam | 76.7 | 84.7 | 83.8 | 85.8 | 84.4 | 80.2 |
| Meropenem | 77.0 | 85.3 | 84.9 | 85.2 | 85.6 | 80.6 |
| Imipenem | 75.2 | 85.5 | 83.3 | 84.8 | 84.2 | 79.3 |
| Ceftazidime | 81.3 | 87.5 | 87.2 | 89.6 | 86.8 | 84.2 |
| Cefepime | 82.4 | 89.9 | 88.6 | 89.3 | 88.2 | 85.3 |
| Levofloxacin | 65.6 | 74.9 | 72.5 | 67.2 | 78.6 | 68.7 |
| Tobramycin | 85.8 | 93.9 | 93.3 | 91.2 | 93.1 | 89.0 |

^a^ Imipenem-relebactam tested only from 2020 to 2024.
^b^ Meropenem-vaborbactam is not approved by the US FDA for treatment of *P. aeruginosa* infections; susceptibility based on EUCAST criteria (susceptible at ≤8 mg/L) [11].

Abbreviations: BSI, bloodstream infection; SSSI, skin and skin structure infection; UTI, urinary tract infection.

**Figure S1**. Frequency of resistant subsets

Abbreviations: MDR, multidrug-resistant; DTR, difficult-to-treat resistant.

Alt text: Bar graph showing DTR and MDR rates stratified by year (2015-2024)
